# The value of stress management programs for medical students: a systematic review

**DOI:** 10.3389/fpubh.2025.1737330

**Published:** 2026-01-13

**Authors:** Khalid A. Bin Abdulrahman, Mohamed Hefny, Saleh Ahmed Alghamdi

**Affiliations:** 1 Department of Medical Education, College of Medicine, Imam Mohammad Ibn Saud Islamic University (IMSIU), Riyadh, Saudi Arabia; 2 Department of Rheumatology & Rehabilitation, Faculty of Medicine, Suez Canal University, Ismailia, Egypt; 3 Department of Psychiatry, College of Medicne, Imam Mohammad Ibn Saud Islamic University (IMSIU), Riyadh, Saudi Arabia

**Keywords:** coping mechanisms, medical students, stress, stress management, workshops

## Abstract

**Context:**

Managing stress carries significant implications for the mental wellbeing, academic achievements, career advancement, and overall life satisfaction of medical students. However, the coping mechanisms for stress management are diverse. The current systematic review examines the proposals and consequences of stress management for students in medical education, acknowledging the array of challenges inherent to their academic journey.

**Methods:**

A search was conducted from 2013 to 2023 (September) across databases such as PubMed, Web of Science, and Scopus to retrieve articles on “Medical students,” “Coping mechanisms,” and “Stress management”. The English-language articles were the only ones considered in the study. The articles that discussed coping mechanisms, such as resilience training, mindfulness-based stress reduction (MBSR) programs, and/or wellness and self-care workshops, were included in the study. This review was not prospectively registered in PROSPERO; however, PRISMA 2020 guidelines were followed throughout.

**Results:**

Nine articles were included in the study based on the inclusion and exclusion criteria. Among 2,334 medical students, 11%, 72.7%, and 16.3% reported low, moderate, and high stress levels, respectively. The selected nine articles aimed to address these far-reaching effects of stress management strategies. The stress management programs encompass creating robust mental health support systems within institutions, incorporating anxiety management education into regular coursework, offering workshops that teach practical techniques for handling emotional turmoil effectively, and fostering an environment where discussing one's challenges is encouraged rather than stigmatized. Such programs encourage students to seek expert guidance when needed, undertake systematic investigations to tailor interventions to changing needs and circumstances, and emphasize the importance of balancing work responsibilities, academic commitments, and personal life.

**Conclusions:**

By implementing these suggestions within their framework, institutions can more effectively prepare their medical learners to overcome educational challenges and cultivate empathetic, resilient professionals skilled in delivering healthcare services.

## Highlights

The stress management programmes involve establishing strong support structures for mental health treatment within institutions:

- Integrating education on anxiety management into regular coursework;
- Offering workshops that provide practical strategies for managing emotional distress;
- Promoting a culture that encourages students to seek expert help when necessary;
- Conducting ongoing investigations to tailor interventions maintaining a harmonious equilibrium between work or academic obligations and personal life.

## 1 Introductions

Embarking on a journey in medical academia is an intriguing yet challenging pursuit marked by its unique blend of scholarly intensity, hands-on clinical exposure, and a deep-rooted dedication to the art and science of remedial care (1). The goal of becoming a medical professional is admirable, but the path to achieving it is paved with numerous obstacles. Many of these challenges are intimately tied to an overarching theme—stress (2, 3). The issue concerning how medical students manage stress deserves thorough investigation. This subject envelops vital aspects like medical education standards and student wellness, eventually influencing healthcare delivery quality (4, 5).

In the sphere of advanced education, medical students hold a unique position. Their learning process is a blend of intellectual rigor and practical clinical experiences, firmly grounded on medical science tenets and an unshakeable adherence to healthcare ethics (6, 7). The curriculum in medicine is renowned for its stringent and comprehensive content, which spans various fields, from fundamental sciences to hands-on clinical practice, all within a highly condensed timeframe (8, 9). An excessive dedication toward activities once found enjoyable can lead to exhaustion or “burnout” when motivation becomes dysfunctional (10). Modern-day medical students and trainees are reporting potentially elevated rates of burnout. Burnout is a psychosocial state typified by reduced motivation, performance degradation, and deteriorating mental wellbeing (11, 12). These unique characteristics combine to form an academic atmosphere that subjects medical students to constant, intense pressure, molding their experiences, viewpoints, and future paths (5).

Stress is commonly defined as “a state of mental or emotional strain resulting from demanding circumstances,” a concept originating from Hans Selye's foundational work in the 1930s, which described stress as the body's non-specific response to challenges (13). Over several decades, this definition evolved to encompass psychological, behavioral, and environmental components relevant to academic and clinical settings, including medical training (14). Modern perspectives regard stress among medical students as a multidimensional interaction between internal coping capacity and external academic and clinical pressures (15).

Medical students consistently report higher stress, anxiety, and burnout levels than the general population, affecting academic performance, empathy, and patient safety (3, 16). Although numerous stress-management interventions have been introduced, including resilience training, mindfulness-based programs, and institutional wellness initiatives, their effectiveness varies widely, and the long-term sustainability of benefits remains unclear (17).

Apart from the stress related to their academic pursuits, students may also confront tension from diverse areas, such as social interactions, their physical environment, and familial issues (18). The pressures faced by medical students can be broadly categorized into three dominant spheres: challenges in academia, social obstacles, and fiscal crises (19). The root cause of their stress often lies in the colossal amount and intricacy of knowledge they are expected to assimilate. An extensive compendium forms the backbone of a medical curriculum, encompassing aspects such as anatomy, physiology, pharmacology, and clinical data—each element crucial for an effective medical practice. Its voluminous content, complex scientific nuances, and high standards set for performance evaluation create a daunting journey for these learners, requiring constant commitment toward scholarly activities.

The elevated status of the medical field inadvertently escalates the pressure experienced by its students (20). The comprehension they gain is acutely understood to form the foundation of their future professional endeavors, with their decisions and actions potentially impacting patient outcomes (21). This intrinsic seriousness associated with medicine imparts a unique nature to the stress borne by these students. They carry on their shoulders the potential responsibility for lives that may be entrusted to them in due course (22).

The practical aspects of medical pedagogy add a layer of intricacy. Critical facets of medical instruction, such as clinical rounds, patient interfacing, and the tangible attributes of healthcare provision, are integral. These encounters play a vital role in career growth and impose various pressures. Medical trainees must navigate the challenges inherent in their clinical duties, manage the emotional toll incurred by exposure to disease and hardship, and develop skills for effective interaction with patients and fellow healthcare providers. These elements collectively contribute to the elaborate stress tapestry that pervades their scholarly existence (23).

Various strategies are essential for medical students to manage stress and maintain their wellbeing (24). Such strategies are available, including problem-centered, emotion-centered, and social support methods (25). Problem-centered coping involves proactively identifying the source of stress and taking measures to rectify the issue (26, 27). On the other hand, emotion-based coping emphasizes emotional regulation coupled with stress management through relaxation practices, mindfulness exercises, and self-nurture (28). A social support strategy involves reaching out to friends or family members or seeking guidance from mentors when encountering challenging circumstances (29). Research has revealed several commonly employed strategies among medical students. The terminologies “active adaptation,” “positive reinterpretation,” “formulating plans,” “conciliation,” and “pursuing social backing” are all incorporated into this category (30, 31).

Despite growth in literature, current research lacks clarity in several areas:

- Limited comparative evaluation of different stress-management approaches across diverse cultural and educational environments (15).
- Limited longitudinal studies examining sustained changes in coping and wellbeing beyond the intervention period (32).
- Insufficient evidence identifying which components of resilience, mindfulness, or wellness programs produce the strongest outcomes (17).
- Few systematic analyses focus specifically on undergraduate medical students during different stages of training (2).

Since the rates of stress and anxiety among medical students are growing steadily, effective programs and interventions must be provided to enhance the mental health of all students to reduce this phenomenon, which is widespread in most medical schools worldwide. This is why medical colleges have developed programs to promote mental health and address the psychological pressures that students face while pursuing a career in medicine. Despite the great diversity of mental health promotion programs available in medical schools and higher educational institutions, including group self-development, yoga and relaxation therapy, mindfulness and lifestyle improvement programs, stress management programs, time management programs, meditation sessions, etc. It aims to help students cope with stress and manage school and life pressures effectively; however, its effectiveness and positive impact on students' mental health are uncertain. This study aims to measure the impact and effectiveness of stress management and coping programs provided to medical students.

## 2 Methods

### 2.1 Study design

A thorough examination was conducted on all research papers related to stress management in medical education for potential inclusion. This scrutiny aims to evaluate the merit of these reviews as impartially as feasible. Quality inspection encompasses factors such as study design, sampling dictates (size and method), gauged outcomes, and data reliability and accuracy. Additionally, an assessment regarding the strictness adhered to in these chosen studies was conducted by authors based on aspects like availability and caliber of study design, study sampling procedures followed, response rate obtained from participants or respondents involved in each respective study, choice of measurement tool employed throughout each research project, along with their specific stress management program.

### 2.2 Search sources and strategies

The search process was conducted systematically using four major electronic databases: PubMed, Scopus, Web of Science, and MEDLINE. The search employed combinations of the following key terms: “*medical students,” “coping mechanisms,”* and “*stress management,”* integrated with Boolean operators appropriate for each database.

The search was carried out between 1 and 5 September 2023, covering peer-reviewed publications published from January 2013 to September 2023. Titles and abstracts retrieved through the initial search were screened for relevance, and full-text articles were obtained when eligibility could not be determined from the abstract alone. All potentially eligible articles were read in full before a final inclusion decision was made.

In addition to the electronic database searches, we performed manual screening of the reference lists of all included articles to identify any additional relevant studies. Gray literature, including conference abstracts and dissertations, was not included due to inconsistent methodological quality.

Data extraction forms were used to collect standardized information from included studies. All procedures adhered to the PRISMA 2020 guidelines, ensuring transparency, rigor, and reproducibility in reporting the review process (33).

### 2.3 Inclusion and exclusion criteria

Studies were eligible for inclusion if they examined stress management or coping interventions—including but not limited to resilience training, mindfulness-based stress reduction (MBSR) programs, and wellness or self-care workshops—among undergraduate medical students from the first to final year of training. Only studies in which data for undergraduate medical students were independently extractable were included; in studies involving mixed health-discipline samples, only outcomes specific to medical students were considered.

Eligible study designs included randomized controlled trials, non-randomized interventional studies, cross-sectional studies, qualitative studies, and quasi-experimental designs. Articles were required to report empirical data related to stress levels, coping mechanisms, or the outcomes of stress-management interventions.

Studies were limited to peer-reviewed articles published in English between January 2013 and September 2023. Articles published in languages other than English were excluded. Restricting the search to English-language publications may have introduced language bias; this limitation has been acknowledged in the Discussion section.

Two reviewers independently screened titles, abstracts, and full texts. Disagreements were resolved through discussion, and when needed, by consulting a third reviewer.

Each included study underwent a formal quality appraisal using the Joanna Briggs Institute (JBI) critical appraisal tools appropriate for its design. Overall, study quality ranged from moderate to high; no study was excluded based on risk-of-bias assessment.

A comprehensive summary of all included studies, including design, setting, sample characteristics, intervention description, and outcomes, is presented in Table 1.

**Table 1:** List of selected articles for the study.

**Reference**	**Country**	**Study design**	**Study samples**	**Academic year**	**Stress level (%)**			**Stress management program**	**Outcomes**
					**Low**	**Moderate**	**High**		
Donohoe et al. (34)	Ireland	Non-RCT observational study	98 (*n* = 204)	Third-year medical students	–	–	–	Resilience- promoting program	Enhanced emotional and mental wellbeing
Daya et al. (35)	India	Cross-sectional study	446	First-year to final year	56.50	32.73	10.76	Perceived stress scale (PSS)	Moderate stress level, showing stress-handling abilities
Bodys-Cupak et al. (36), Donohoe et al. (34)	Poland	Piolet study	526	First-year to final-year students of nursing, physiotherapy, medical rescue, and obstetrics	21.00	29.18	49.82	PSS	Greater sense of self-efficacy who chose active stress coping strategies
Teh et al. (37)	Malaysia	Cross-sectional study	371	First-year to final year	63.00	21.60	15.40	Resilience program, which includes Rosenberg Self-Esteem Scale (RSES), Rosenberg Self-Esteem Scale (RSES), Brief COPE	Reduce stress and sustain wellbeing
Rafique (38)	Pakistan	Cross-sectional study	125	Medical students	–	–	–	Resilience strategy	–
Sahlabadi et al. (39)	Iran	Cross-sectional study	325	Undergraduate medical students (pursuing nursing, paramedical, and physiotherapists)	1.00	31.00	66.30	Stress score and Osipow questionnaires, followed by content validity rate (CVR)	Work and study environments demand stress management programmes
Abouammoh et al. (40)	Saudi Arabia	Qualitative study	33	Final-year medical students, medical interns	–	–	42.42	Stress coping strategy	Medical students and interns struggle to lead healthy stress-coping strategies
Abdul Aziz et al. (41)	Malaysia	Cross-sectional study	290	Undergraduate medical students	9.00	73.40	17.60	PSS and multidimensional scale of perceived social support (MSPSS)	Social support from family is the strongest for students to go through the stress of tough times
Aisha (42)	Larkana	Quasi-experimental study	120 (*n* = 240)	First-year	13.00	72.00	15.00	2-day workshop on stress management	The workshop was helpful and improved memorisation, time management, and self-directed conflict management
Total	–	–	2,334	–	11	72.7	16.3	–	–

–, Not available.

Outcomes were harmonized by grouping results into standardized domains (stress levels, intervention effects, and coping mechanisms), allowing clearer comparison across studies.

Where reported by the original studies, effect sizes, pre–post comparisons, or percentage changes were extracted and added to the synthesis.

## 3 Results

**Figure 1:**
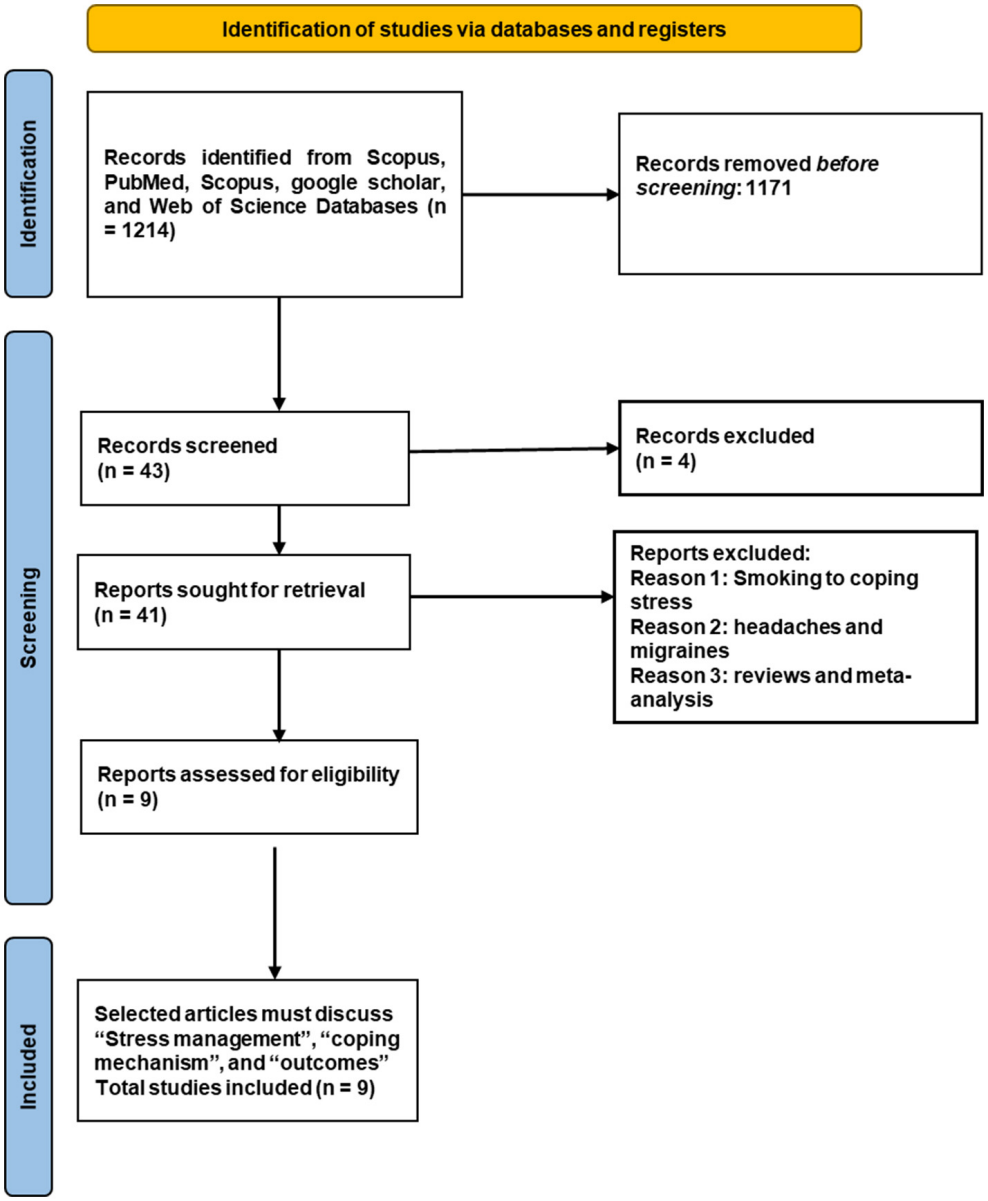
PRISMA flow chart.

An overview of the literature selection process is shown in Figure 1. We evaluated 43 papers from existing literature sources against our selection criteria.

Upon additional scrutiny and abstract perusal, we focused on nine articles to gain insight into the resilience techniques employed by medical students (Table 1). It was noted that, among the nine selected articles, five were cross-sectional studies, while the others were qualitative, observational, pilot, and quasi-experimental studies.

In a non-randomized observational study conducted in Ireland, Donohoe et al. (34) observed the effects of a program designed to bolster resilience among third-year medical students. The outcomes of their research indicated an improvement in emotional and mental health among the participants. These findings suggest that interventions designed to enhance resilience can positively impact the psychological wellbeing of medical students, potentially enabling them to manage stress more effectively. The research conducted by Daya et al. (35) incorporated participants from multiple academic years in a cross-sectional analysis within India to evaluate their perceived stress levels. Moderate to high levels of stress, as measured by 32.73 and 10.76%, were observed among first- to final-year medical students. The results indicated a median stress level among students, while also revealing their capacity to manage such pressures. This suggests that despite the stress factors among students, they appear to have developed efficient strategies to counteract this adversity. The preliminary research conducted in Poland engaged students pursuing diverse health-related fields and suggested that the students who opted for active stress-management techniques exhibited a heightened sense of self-belief. This emphasizes the significance of encouraging students to embrace active methodologies for handling stress, enhancing their sense of self-efficacy (36). In the same study, high levels of stress, at approximately 49.82%, were observed among medical professionals pursuing nursing, paramedical, and physiotherapist courses. In a comprehensive research study conducted in Malaysia, an initiative to boost resilience was introduced to students across diverse academic levels, where approximately 63.0%, 21.6%, and 15.4% of students were found to have low, moderate, and high-stress levels, respectively. The outcome demonstrated a significant reduction in stress and the preservation of wellness. This suggests that initiatives aimed at enhancing resilience may serve as efficient tools for augmenting the mental health status of students (37).

Upon evaluating approximately 125 Pakistani medical professionals with 5–10 years of teaching experience, it was observed that junior faculty members often exhibit behavioral problems due to high stress levels (38). Another study from Iran by Sahlabadi et al. (39) noticed that 66.30% of undergraduate students possessed high stress levels, making them 11.829% times more likely to have suicidal thoughts. Abouammoh et al. (40) noted that 42.42% of medical final-year and internship students experienced stress and employed stress-coping strategies, including avoiding medical discussions, smoking, and engaging in physical exercise, which helped them cope with stress. A study conducted by Abdul Aziz et al. (41) among 290 undergraduate medical students suggested that 73.40 and 17.60% of students possessed moderate and high stress levels, respectively. The study highlighted that social support from family managed the stress among these students. A study by Aisha (42) found moderate and high-stress levels in 72 and 15% of first-year medical students from Pakistan, respectively. The most common stressors among these students were frequent exams, inadequate time management, insufficient teaching guidance, and difficulty in memorizing.

The research highlights the diverse methods employed in stress management programs and their effects on students from various countries and educational backgrounds. The findings underscore the importance of customized interventions and support networks that help students manage stress effectively, thereby promoting their psychological wellbeing. Both proactive coping mechanisms and interpersonal assistance are pivotal in mitigating stress among students.

## 4 Discussion

Medical education exposes learners to sustained academic, clinical, and emotional stressors. These findings align with international studies showing that medical students consistently experience high levels of psychological distress during all stages of training (2, 15). The results of this review confirm that moderate to high stress is prevalent worldwide and underscore the importance of structured interventions.

1. The path of medical education is a demanding and strenuous journey, fraught with numerous pressures that test the mettle of even the most tenacious individuals. To tackle these obstacles and promote their wellbeing, aspiring physicians employ various approaches to manage stress. These techniques are instrumental in preserving their psychological and emotional wellbeing amid the strict requirements of medical training. The following discussion provides an in-depth exploration of these stressors and stress management methods. Evidence strength varied substantially across designs; heterogeneity in measurement tools, intervention types, and follow-up duration limits comparability. Significant gaps exist in randomized evaluations and long-term outcomes.

### 4.1 Sources of stress among medical students

The field of medical education, known for its stringent requirements and complex study modules, presents a unique assortment of stress-inducing factors that are distinctly different from those found in other academic endeavors. Identifying the precise causes of stress among students pursuing a career in medicine is crucial, as it enables the formulation of effective strategies and support systems to promote their overall wellbeing. Previous research indicates that the incidence rate of stress among medical students fluctuates between 30 and 50% (43, 44). Compared with the general population or students in other academic disciplines, this stress level is notably elevated (44, 45).

### 4.2 Academic pressures

The demanding academic workload in medical education is a significant source of stress for students, who must master extensive scientific and clinical content under strict deadlines (4, 9, 46). Studies consistently show higher academic stress among female students, potentially due to additional social responsibilities, greater exam-related pressure, and biological factors influencing stress responses (44, 47, 48). However, some reports note slightly higher stress levels among males in certain contexts (49).

High-stakes examinations, competition for strong academic standing, and the pursuit of residency placements further intensify performance pressure (50, 51). Common stress manifestations include restlessness, anxiety, somatic symptoms, and maladaptive coping behaviors, with many students unable to identify effective stress-management strategies (44). Additional factors, such as obesity, have also been linked with heightened stress (52).

Impostor syndrome—characterized by self-doubt and fear of inadequacy—is another contributor to academic stress, often exacerbated by the highly competitive environment of medical education (53).

### 4.3 Clinical rotations

The participation of medical scholars in clinical rounds, which includes patient care and hospital supervision, infuses a new level of tension into their academic lives (54). Although essential for career growth, these practical activities carry their unique stressors. For novices stepping into this realm for the first time, the obligation to make informed clinical judgments, perform physical assessments, and interact effectively with patients and healthcare teams can be overwhelming. Various research findings have drawn connections between increased clinical duties during the last years of medical academia and a rise in stress levels (47, 55).

Medical trainees who serve as healthcare professionals are at an increased risk of contracting COVID-19 due to their higher exposure. Apprehensions surrounding potential infection during clinical rounds serve as additional stress inducers for these students (56). Contemporary studies indicate that medical students have reported clinically significant psychological distress and decreased mental health conditions (57). Furthermore, they presented deteriorated sleep patterns and reduced appetite compared to pre-pandemic scenarios (58).

The kind of rotation experienced during medical school could play a pivotal role in the occurrence and intensity of sleep deprivation throughout the study years (59). Rotations involving surgery or emergency medicine tend to be more grueling, leading to increased sleep deprivation compared to rotations through departments with fewer demands. Physiological issues such as sleep apnea also contribute significantly to sleep disturbances. Research on identifying risk factors for sleep apnea among Pakistani medical students revealed that disruptive snoring was present in 27% of male and 12% of female participants (60). Additionally, the erratic schedules accompanying clinical rotations disrupt students' sleep routines and interfere with their personal lives, thereby escalating overall stress levels. The struggle to balance clinical tasks and academic obligations further heightens the complexity associated with their roles. Sleep has been critical to stabilizing and enhancing cognitive processes (60).

Cognitive competencies such as consolidating and encoding are critical in higher education contexts, particularly in medical training. This is because medical students must grasp vast amounts of intricate factual information (61). An investigation involving Brazilian medical students revealed that excessive daytime sleepiness negatively influenced their academic output (62). This lack of sufficient rest results in increased drowsiness during subsequent nocturnal work hours, especially in the latter half of the period. The consequence is an increased propensity for mistakes and accidents on duty and while commuting home, for instance, falling asleep while driving (63). The level of self-awareness regarding sleep and general understanding appears to be insufficient among many of the analyzed groups; consequently, enhancing knowledge among students could be advantageous. Tailoring student schedules based on individual circadian rhythms may also bring benefits (60).

### 4.4 High expectations and perfectionism

The healthcare field is characterized by an environment of high standards and a drive toward perfection, which can serve as both a source of inspiration and strain for those studying medicine (64). This eagerness to reach or surpass such benchmarks often becomes a self-driven demand to thrive in educational and clinical settings, resulting in apprehension about failing or not meeting the assumed proficiency levels. Perfectionism is a multifaceted personality trait with both beneficial and harmful subcategories (65). Henning's team conducted research on psychological adaptation among students from various health disciplines. They concluded that perfectionism and the impostor phenomenon (IP) were significant predictors of psychological discomfort among medical students (64, 66).

The IP is predominantly found in professions where intellectual prowess is highly valued, such as academia. Individuals attracted to these fields often showcase perfectionistic tendencies and function in a goal-oriented approach (67). IP reports are prevalent in all hierarchies, ranging from novice students to seasoned surgeons. As healthcare professionals are plagued by burnout and its damaging implications, impostor syndrome significantly adds to this problem, making it crucial to deepen our understanding of this occurrence (68, 69). The anxiety arising from potential medical inaccuracies or an incorrect diagnosis due to their severe implications contributes to medical students' stress. This quest for flawlessness can weaken your self-belief and further intensify stress levels.

Self-cared perfectionism encapsulates a range of traits, such as the tendency to set excessively high goals and participate in rigorous self-assessment. On the other hand, other-centered perfectionism revolves around establishing unattainable standards for others and critiquing them harshly (70). This personality type begins to associate success with feelings of anxiety and nervousness while attributing their accomplishments to external factors such as fortuitousness. However, the satisfaction derived from these achievements is fleeting as they face the upcoming challenge, thus perpetuating this cycle (71). In addition, numerous research endeavors have corroborated that IP and perfectionistic tendencies are substantial indicators of emotional turmoil among medical students.

Furthermore, these studies suggest that an inclination toward perfectionism predicts susceptibility to disorders such as anxiety and depression (72). The correlation between IP and perfectionism introduces an added dimension when delivering or receiving criticism or feedback. Those affected by IP typically form skewed reference frames for self-evaluation, whereby successes are not acknowledged internally, thus inhibiting effective feedback mechanisms (73). The variance in interpretations when it comes to accepting input from others underpins the need for those who offer feedback to pay heedful attention during exchanges.

In summary, the origins of tension among medical students are complex and diverse, deeply embedded within the unique requirements and expectations of their medical academic journey. These sources of stress include rigorous academic demands, clinical obligations, striving for perfectionism, struggles with balancing personal life and work commitments, financial worries, competition amongst peers, and adaptation challenges. A critical move toward ensuring comprehensive wellbeing for medical students is acknowledging this complex web of stressors. This recognition highlights the need for personalized strategies and supportive frameworks designed to identify these pressures while minimizing their impact within the distinct environment of medical education.

### 4.5 Stress management programs

#### 4.5.1 Resilience training

Resilience training programs are designed to equip medical learners with the psychological and behavioral skills required to withstand and recover from the inherent pressures of medical education and clinical practice. The theoretical foundation of resilience in medical education draws heavily from cognitive-behavioral theory, stress-adaptation models, and positive psychology. These frameworks conceptualize resilience as a dynamic process involving adaptive coping, cognitive reframing, emotional regulation, and the ability to derive meaning from stressful experiences. In the context of medical training, resilience is increasingly viewed not as an innate trait but as a set of teachable, modifiable skills that enable students to respond constructively to academic, clinical, and interpersonal challenges.

From a cognitive-behavioral perspective, resilience training emphasizes identifying unhelpful thought patterns, reframing stressors, and developing more effective problem-solving approaches. Positive psychology contributes the principles of growth mindset, self-efficacy, and reflective meaning-making, all of which are essential for sustaining wellbeing in demanding learning environments. These principles help medical students shift from a deficit-focused mindset (i.e., stress as harmful) to a competence-enhancing one (i.e., stress as an opportunity for growth).

In practice, resilience curricula often include workshops, guided reflection sessions, peer discussions, and experiential activities designed to strengthen adaptability, emotional awareness, and stress tolerance. As noted in North American medical education programs, resilience modules are typically structured to promote autonomy and self-directed learning—key characteristics of adult learning theory (74, 75). Reflection periods, an integral component of these programs, encourage students to analyze clinical encounters, emotional responses, and performance challenges, thereby reinforcing self-regulation and metacognitive awareness (76).

Resilience training compares favorably with other stress-management approaches identified in the included studies—particularly mindfulness-based stress reduction (MBSR) and general coping-skills workshops. While MBSR focuses on present-moment awareness, non-judgmental observation of thoughts, and physiological relaxation, resilience programs target cognitive flexibility, proactive coping, and long-term adaptability. Both approaches demonstrate benefits for stress reduction; however, resilience training may offer broader applicability because it equips students with cognitive and behavioral tools that are useful across varied clinical and academic contexts. Unlike brief coping-skills programs that focus on isolated strategies, resilience interventions promote sustained behavioral change and the development of internal resources that persist beyond the intervention period.

Evidence from the reviewed studies suggests that resilience training is particularly effective when students actively engage in structured reflection, open discussions about stress and medical errors, and collaborative problem-solving. Students who adopt proactive coping styles—such as seeking support or reframing stressful events—tend to report lower burnout levels and better psychological wellbeing compared to those who rely on avoidance strategies (77, 78). Programs such as the Penn Resiliency Program (PRP), grounded in cognitive-behavioral therapy, have shown significant reductions in depressive symptoms and improvements in emotional functioning across diverse educational contexts (79).

Based on the findings from this review and supporting literature, several evidence-based recommendations can curricula into medical programs:

1. Embed resilience training longitudinally across the curriculum, allowing students to practice and reinforce skills at multiple stages of training rather than in isolated workshops.
2. Incorporate structured reflection and debriefing sessions, especially after stressful clinical encounters, to promote emotional processing and self-awareness.
3. Combine resilience modules with MBSR or wellness programs, as multimodal approaches appear to strengthen overall intervention effectiveness.
4. Create safe, non-judgmental discussion spaces where students can openly share experiences related to stress, burnout, and clinical challenges, fostering peer support networks.
5. Train faculty mentors in resilience-supportive practices, enabling them to model adaptive coping behaviors and provide effective guidance.
6. Ensure institutional support, recognizing that resilience is not solely a student responsibility; organizational culture, workload, and learning environments play crucial roles in shaping resilience.

In summary, resilience training offers a comprehensive approach to stress management by simultaneously targeting cognitive, emotional, and behavioral mechanisms. When embedded within a supportive institutional framework, resilience programs can significantly improve medical students' coping capacity, mental health, and long-term professional resilience.

#### 4.5.2 Mindfulness-based stress reduction (MBSR) programs

MBSR-based programs introduce learners to practices centered on mindfulness, including meditation and other exercises that employ a mindfulness-based approach. These methodologies aim to equip students with tools for better stress management, improved focus, and promoting emotional health. Often, MBSR programs include sessions focused on cultivating mindfulness and promoting the regular practice of this discipline as part of self-care routines among students. Kabat-Zinn's model, widely recognized as the quintessential guide for a mindfulness course, is typically presented as an 8-week program with weekly classes known as the MBSR program (80).

Innovative strategies are essential for enhancing the accessibility of mindfulness meditation exposure to medical students. A preliminary investigation by Danilewitz et al. (81) examined a strategy led by peers in the context of medical student mindfulness, yielding substantial stress reduction and notable enhancements in self-compassion, altruism, and mindfulness (81). This approach, led by peers, offers considerable economic advantages while potentially fostering enhanced course comfort, given that it is delivered not by authority figures but fellow students. An introductory mindfulness session was offered alongside an 8-week course designed specifically for physicians, introducing a potential clinical therapeutic modality and self-care system. A more intensive experience, that is, participating fully in the course rather than attending just one introductory session, was associated with increased comfort levels and a higher likelihood of adopting mindfulness modalities (82).

A well-structured method, MBSR (Mindfulness-Based Stress Reduction), has been established to teach mindfulness principles within scientific circles (83). The evidence further supports its utility in academic settings geared toward healthcare professions. While it is rational to anticipate some variation when executing any MBSR protocol, the fundamental components and overarching framework must stay broadly consistent. The original curriculum was designed as a regimen spanning 8 weeks, involving weekly sessions lasting 2 h each, supplemented by daily exercises at home and a single-day retreat (83).

In their progressive analysis, Erogul and his team identified a persistent inclination toward reduced perceived stress over a 6-month period, albeit without statistical significance. Similarly, Aherne and his colleagues discovered through qualitative thematic scrutiny that stress regulation contributed to student satisfaction with the MBSR program (84, 85). These insights underscore the impact of MBSR on managing medical student stress levels and suggest a potential long-term advantage gained from brief engagement with MBSR (86). It is essential to explore this field more, as burnout is crucial to examine, given its correlation with professional conduct, empathy, and patient safety (86, 87). Recognizing how burnout can impact both patient outcomes and wellness among medical students necessitates more in-depth research to accurately determine if there is a connection between MBSR training and instances of burnout.

#### 4.5.3 Wellness and self-care workshops

Programs focused on health promotion and personal care accentuate the need to maintain physical and emotional wellness (88). These initiatives provide students with suitable tactics to incorporate self-care habits into their daily routines, including guidance on nutrition, physical activity, sleep patterns, and relaxation techniques. Workshops could also highlight the importance of balancing professional duties with personal life. Occupational stress can negatively affect the competence, service quality, delivery efficiency, and comprehensive quality of life of healthcare professionals. Thus, pinpointing these occupational hazards and finding ways to alleviate them is crucial in safeguarding the mental stability and general wellness of those involved in patient care services (89).

In educational culture, students have proposed improving communication practices and fostering a more respectful and supportive environment among peers and faculty. Specifically, the implementation of robust mentoring initiatives was encouraged as a means of providing more significant support. Mentorship programmes within medical education contexts have been linked with elevated or improved student academic achievements, facilitating relationships between students and faculty that spur personal growth alongside professional development (90, 91). In addition, the students advocated for reducing the stigma associated with mental health care while expanding access to therapeutic counseling services (88).

Clusters of self-care activities have been classified based on brainstorming responses from medical students, thus providing a more profound comprehension and enhancement of self-care, as per certain research studies (92). A notable decrease in severe depression, stress, and anxiety among medical students was observed upon introducing mindfulness training, social gatherings, a pass/fail evaluation system, and decreased contact hours during the preclinical tenure (93). Furthermore, it is significant that personalized self-care strategies are transformed into standard institutional cultural practices aimed at developing resilience. This helps circumvent obstacles while prioritizing self-care among healthcare professionals.

Institutionally, the acknowledgment and integration of self-care practices are vital for safeguarding mental wellbeing, fostering resilience, and enhancing work motivation, job contentment, patient security, and quality of service. The endorsement of self-care techniques, resilience strategies, and workplace policy modifications at an institutional level might incorporate compulsory rest periods (including brief sleep increments or “power naps,” casual strolls, or access to quiet spaces), implementation of short breaks before each meeting's initiation, and regulations mandating meetings always commence on the hour. All these methods aim to refine the professional environment (89).

Recent analyses emphasize the necessity of harmonizing personal needs with those of others, advocating self-care as the primary protective measure for medical professionals grappling with the demands of treating COVID-19 patients, the enduring nature of this crisis, and its impact on daily life habits. In this context, these evaluations underscore the importance of employing supportive instruments and methodologies to address mental health complications and empathy exhaustion prevalent among healthcare professionals. Various recommended prophylactic self-care maneuvers include spiritual observances and calming methods, electronic mental health provisions, and enhancing interpersonal abilities (94, 95). Furthermore, regulations should limit the duration of work shifts, along with provisions for professional mental health assistance programs and peer-assisted support groups. This will facilitate routine debriefing sessions, where experiences can be shared openly. Furthermore, hospitals and other healthcare establishments should implement comprehensive parental and sick leave policies that do not impose undue stress on fellow healthcare professionals. Departments must factor in reserved time slots within their schedules for personal medical consultations, therapy sessions, or medical visits (89).

It is crucial to note that a combination of these initiatives might be adopted by medical institutions, acknowledging the complex character of the stress endured by medical students. In addition, such initiatives are frequently incorporated into the comprehensive medical curriculum, highlighting the importance of mental wellbeing as a fundamental aspect of medical scholars. These initiatives aim to support students in their individual and vocational growth, ultimately priming them to evolve into tenacious and empathetic practitioners in healthcare (96–98).

### 4.6 Implementation and recommendations

The strategies available to support medical students in managing stress are broad in scope and critical to their success. Given the demanding nature of medical training, institutions must prioritize students' psychological wellbeing. Effective stress management enhances mental health, academic performance, and resilience. Key measures include establishing robust mental health support systems, integrating stress management education into curricula, offering coping skills workshops, and promoting an open, help-seeking culture. Ongoing research is also needed to tailor interventions to students' evolving needs. Together, these strategies create a supportive environment that prepares future physicians to thrive academically and develop into compassionate, resilient healthcare professionals.

### 4.7 Limitations

A limitation of the study is the relatively brief follow-up, which may be applicable to interventions such as workshops and resilience programs. Observed outcomes may not be adequately assessed in terms of their long-term effects. Second, the studies' cultural, institutional, and socioeconomic contexts can influence the effectiveness and applicability of stress management programs. Depending on the culture or educational setting, such results may not be universally applicable. The third problem is that some studies do not provide complete information about the academic year. A lack of this information can limit the interpretation of results, as different levels of stress and coping mechanisms may be associated with different stages in the academic journey. Limiting the search to English-language publications may have excluded relevant international evidence, contributing to potential publication and language bias. It is crucial to understand these limitations to interpret the results and consider the applicability of stress management interventions in diverse educational settings.

## 5 Conclusion

Effective stress-management strategies are essential for medical students, given the demanding nature of their training. Prioritizing students' mental wellbeing enhances academic performance, supports resilience, and contributes to the development of balanced and capable future physicians. Key measures include providing accessible psychological support, integrating stress-management education into curricula, offering coping-skills workshops, and fostering an open, help-seeking culture. Ongoing research is needed to tailor interventions to students' evolving needs.

Stress-management programs are not optional additions but vital components of medical education. By investing in these initiatives, institutions help students navigate adversity, build resilience, and grow into compassionate, competent healthcare professionals. As medical training continues to evolve, programs must be regularly refined to support both academic success and long-term emotional wellbeing, ensuring that future physicians are equipped with the skills to care for themselves and their patients.
